# Mapping N- and C-terminals of *Leishmania donovani* tyrosine aminotransferase by gene truncation strategy: a functional study using in vitro and in silico approaches

**DOI:** 10.1038/s41598-020-69512-y

**Published:** 2020-07-27

**Authors:** Santanu Sasidharan, Prakash Saudagar

**Affiliations:** Department of Biotechnology, National Institute of Technology, Warangal, Telangana 506004 India

**Keywords:** Chemical biology, Proteins

## Abstract

Tyrosine aminotransferase (TAT) catalyzes the transamination of amino acids in *Leishmania* sp.. TAT from *Leishmania donovani* has been found to be extremely stable at extreme temperatures and pH conditions. This study was conceived to map the functions of the non-conserved N-terminal and conserved C-terminal domain of TAT. N-terminal (NTAT) and C-terminal (CTAT) domain of TAT was truncated and cloned into the pET28a(+) vector. The truncated proteins were expressed, purified, and biochemically characterized. The *K*_*m*_ of NTAT and CTAT for the tyrosine-pyruvate pair was determined to be 3.468 ± 0.796 mM and 4.581 ± 0.627 mM, repectively. Temperature and pH stability studies found NTAT to be stable like TAT but CTAT was extremely susceptible to temperature and pH changes. Upon docking and simulation for 100 ns, NTAT had lower SASA values. From UV spectroscopic study, PLP bound better to CTAT than NTAT because of the reduced SASA of NTAT. The sensitivity of CTAT was reasoned when the urea denaturation studies showed two-state denaturation which differed from NTAT’s and TAT’s biphasic folding mechanism. From this study, the authors hypothesize that the N-terminal is responsible for PLP stabilization and C-terminal protects the active site from extreme conditions.

## Introduction

Leishmaniasis results in 70,000–1 million cases (WHO, 2018) worldover every year. This disease is caused by around 20 species of *Leishmania* and causes 20,000–30,000 deaths every year. Initially recorded in India, the disease is now prevalent in most of the under-developed and developing countries like Brazil, Afghanistan, Columbia, Iran, few African countries. Due to the prevalence of this disease in developing nations, leishmaniasis is a neglected disease. Leishmaniasis can manifests mainly in three different clinical forms- visceral leishmaniasis, cutaneous leishmaniasis and mucocutaneous leishmaniasis^1^. Another form called post-kala azar dermal leishmaniasis is also gaining importance as a new form in certain countries^2^. Visceral leishmaniasis is commonly called kala-azar in India and is primarily caused by the species *Leishmania donovani* (Kinetoplastida: Trypanosomatidae)*,* named after its founders. The life cycle of *Leishmania* is very complex as it alternates between the host and an insect vector^3^. In the host, the parasite resides inside macrophages and remains immobile (amastigote form). Later on, the parasite moves into the gut of sand fly (Phlebotomine) after a blood meal and metamorphoses into the motile promastigote form. The alternation of the parasite between the host and insect vector causes up-regulation and down-regulation of genes and proteins which retards the prevention and transmission of the disease.

Currently, the treatment involves the use of antimonial compounds, miltefosine, and amphotericin B^4^. Few antibiotics are also used to combat this disease. Improvisation of efficacy by various drug delivery methods have also been employed wherein the liposomal formulation of Amphotericin B is prescribed widely in India^5^. A combination of anti-leishmanial drugs has also been promising, keeping in mind, the exponentially increasing cases of parasite resistance. Relapse of the parasite infection, even after multiple treatments with various drugs and compounds, has been attributed to resistance towards combinatorial drugs, reduced efficacy of miltefosine and renal toxicity of antibiotics like amphotericin B. These prevailing conditions have forced the need for new drugs and drug targets in the fight against *Leishmania* sp.^6^. Therefore, this study was conducted to understand a new drug target, called tyrosine aminotransferase (TAT), in *Leishmania donovani.*

Tyrosine aminotransferase (L-tyrosine: 2 oxoglutarate aminotransferase; EC 2.6.1.5; TATase) is an enzyme that is widely distributed in all organisms. The enzyme is pyridoxal-l-phosphate (PLP) dependent for its catalytic activity. TAT has been elucidated to be a homodimer from previous studies and belongs to the fold type I aminotransferase^7,8^. The role of the enzyme is to metabolize tyrosine in the cell and transfer the amino group to a co-substrate. The enzyme activity has been attributed to the pathogenesis in other trypanosomatids like *Trypanosoma brucei.* The end products of this enzyme reaction have been known to increase pathogenicity in *Trypanosoma brucei.* The depletion of amino acids reserves in the *Leishmania* parasite will lead to irregularities in the parasite metabolism and survival. The negative effect of the end products and the role of re-oxidation of nicotinamide adenine dinucleotide (NADH) by the enzyme encouraged the authors to choose this enzyme for characterization. The mechanism of tyrosine aminotransferase activity involves the conversion of pyruvate to alanine. The by-product is excreted out of the parasite in *Trypanosoma cruzi.* Other by-products of the reaction like 4-hydroxyphenyl pyruvate are reduced to aromatic lactates through a series of sequential reactions catalyzed by dehydrogenases. These dehydrogenases are involved in the re-oxidation of NADH in the cytoplasm of various parasites. These NADHs are later involved in the Kreb’s cycle compensation and the respiratory chain pathway. In mammals, the TAT enzyme catalyzes a similar reaction to maintain the concentration of tyrosine below the toxicity levels and this enzymatic reaction involves α-ketoglutarate as a co-substrate. In *Leishmania,* the reaction catalyzes the transfer of an amino group to pyruvate. The enzyme TAT also catalyzes various other co-substrates like 2-keto-3-methyl-valerate and α-ketomethiobutyrate^9^. Eventually, the amino acids, methionine, and valine, which are essential amino acids, play an important role in the survival of the parasite in its dimorphic cycle.

From our previous study, we had observed the highly stable nature of tyrosine aminotransferase in *Leishmania donovani*^9,10^*.* Moreover, tyrosine aminotransferase from mouse has also shown temperature and pH stability^11^. Sobrado V. R. et al. mutated the gene sequence of tyrosine aminotransferase of *Trypanosoma cruzi* and found that mutation of Asn54 and Asn57 lowered the catalytic efficiency of tyrosine aminotransferase in *T. cruzi*^12^. The gene sequence of the enzyme in *Leishmania donovani* has a unique N-terminal sequence and a conserved C-terminal sequence. Therefore, the authors decided to map the function of the N-terminal and C-terminal domains of TAT from *Leishmania donovani* by truncating the 60 amino acid residues of both N-terminal (NTAT) and C-terminal (CTAT) regions individually. The genes were cloned, expressed, and purified with the help of molecular biology techniques and Ni–NTA affinity chromatography. The NTAT and CTAT proteins were then biochemically characterized to determine the apparent *K*_*m*_ and *V*_*max*_. The PLP binding studies were conducted by the UV spectroscopy method and furthermore, the folding mechanisms, employing urea denaturant, were also determined with the help of fluorescence spectroscopy. The crystal structure of tyrosine aminotransferase in *Leishmania infantum* and *Trypanosoma cruzi* have been recorded earlier^7,8^. Docking and molecular dynamics simulation studies on NTAT, CTAT, and TAT showed the interactions of PLP with truncated proteins at the molecular level. These experiments, backed by in silico analysis, aims to map the function of the N-terminal and C-terminal domains of tyrosine aminotransferase from *Leishmania donovani*.

## Results

### Sequence analysis

**Figure 1:**
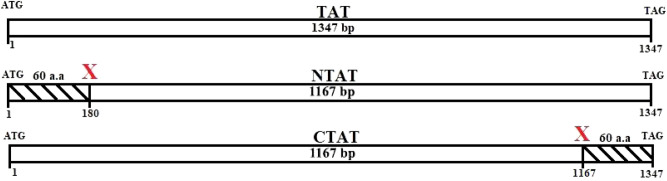
Deletion scheme of NTAT and CTAT: The schematic representation of deletion of 60 amino acids or 180 bp at the N-terminal and C-terminal is shown in the figure. The wild type gene of TAT is also represented in the figure for reference. The final gene size for both the truncated genes was estimated to be around 1,167 bp.

**Figure 2:**
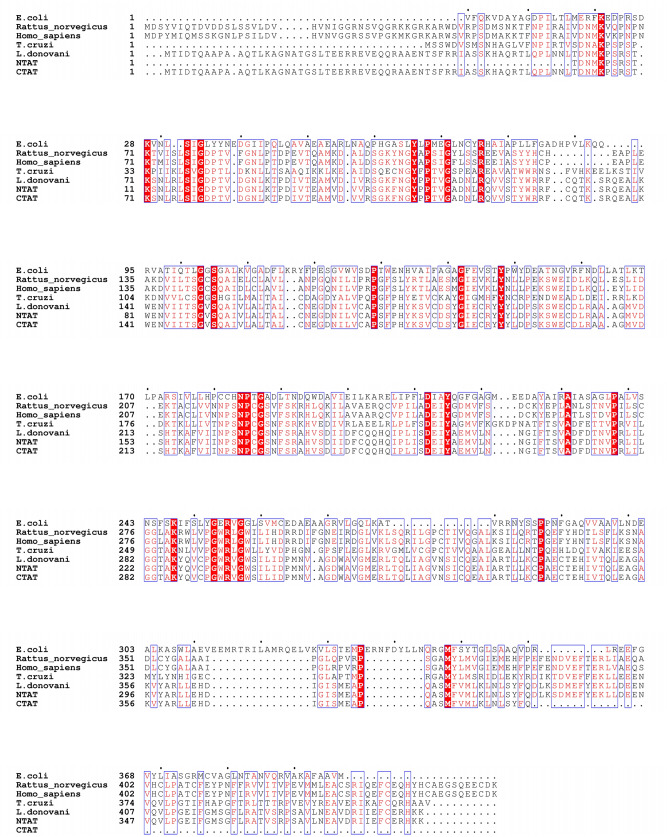
Multiple Sequence alignment analysis: The figure shows the sequence alignment of tyrosine aminotransferase of *L. donovani* with *E. coli* (18.66% identity), *Rattus norvegicus* (35.70% identity), *Homo sapiens* (35.01% identity) and *T. cruzi* (43.66% identity)*.* It is observed that the N-terminal of TAT from *L. donovani* has a very little identity with other TAT sequences but the C-terminal shows a large number of identical sequences conserved across the organisms. The red highlighted boxes with residues in white font depict identical and conserved amino acid residues whereas the blue boxes that are non-highlighted represent similar amino acid residues in red font.

The representation of NTAT and CTAT in Fig. 1 depicts the deletion of 180 bp encompassing 60 amino acids from the native TAT sequence. The truncation of 60 amino acids brings the protein size of NTAT and CTAT to 42.79 kDa approximately, with 389 amino acids each. Clustal Omega analysis results are shown in Fig. 2. The TAT sequence from *E. coli* exhibited 18.66% identity with TAT from *Leishmania donovani* while *Rattus norvegicus* and *Homo sapiens* displayed 35.10% and 35.01% identity respectively. A close relative of *Leishmania* parasite is *Trypanosoma cruzi* that shares 43.66% identity towards the nucleotide sequence of *Leishmania donovani* TAT. The N-terminal domain of LdTAT was found to be very different from the N-terminals of tyrosine aminotransferase of other organisms. The only conserved residues in the first 60 amino acids being I44, S47, Q55, P56, L57, and L60. Structurally, the N-terminal is composed of three α-helix and coils (Supplementary Data 3A,C). The N-terminal domain of *L. donovani* shared only 10% similarity with *Rattus norvegicus, Homo sapiens,* and *T. cruzi. E. coli* has a unique N-terminal sequence like LdTAT and did not show any particular alignment except for the D11, P12 and I13 residues. The active site or the anchoring residue K286 is conserved in all the organisms. When the C-terminal domain was analyzed, there were many conserved residues among the organisms (48% identity in 60 amino acids) like D390, S393, D394, M395, E396, F397, E399, K400, L401, L402, E404, V407, L410, P411, F415, F420, R422, A423, V425, S426, P428, V431, E434, A435, R438, I439, F442, C443 and H446. These amino acid residues constitute three α-helixes and a β-sheet in the C-terminal domain of TAT structure (Supplementary Data 3B,C). These residues were conserved in all the organisms considered and interestingly, the N and C-terminal domains of LdTAT matched considerably well with its close relative *Trypanosoma cruzi. E. coli* being a prokaryote possesses a dissimilar N and C-terminal sequence but, the active site and the residues surrounding the active site residues were related to other organisms.

### Cloning, expression, and purification of NTAT and CTAT

**Figure 3:**
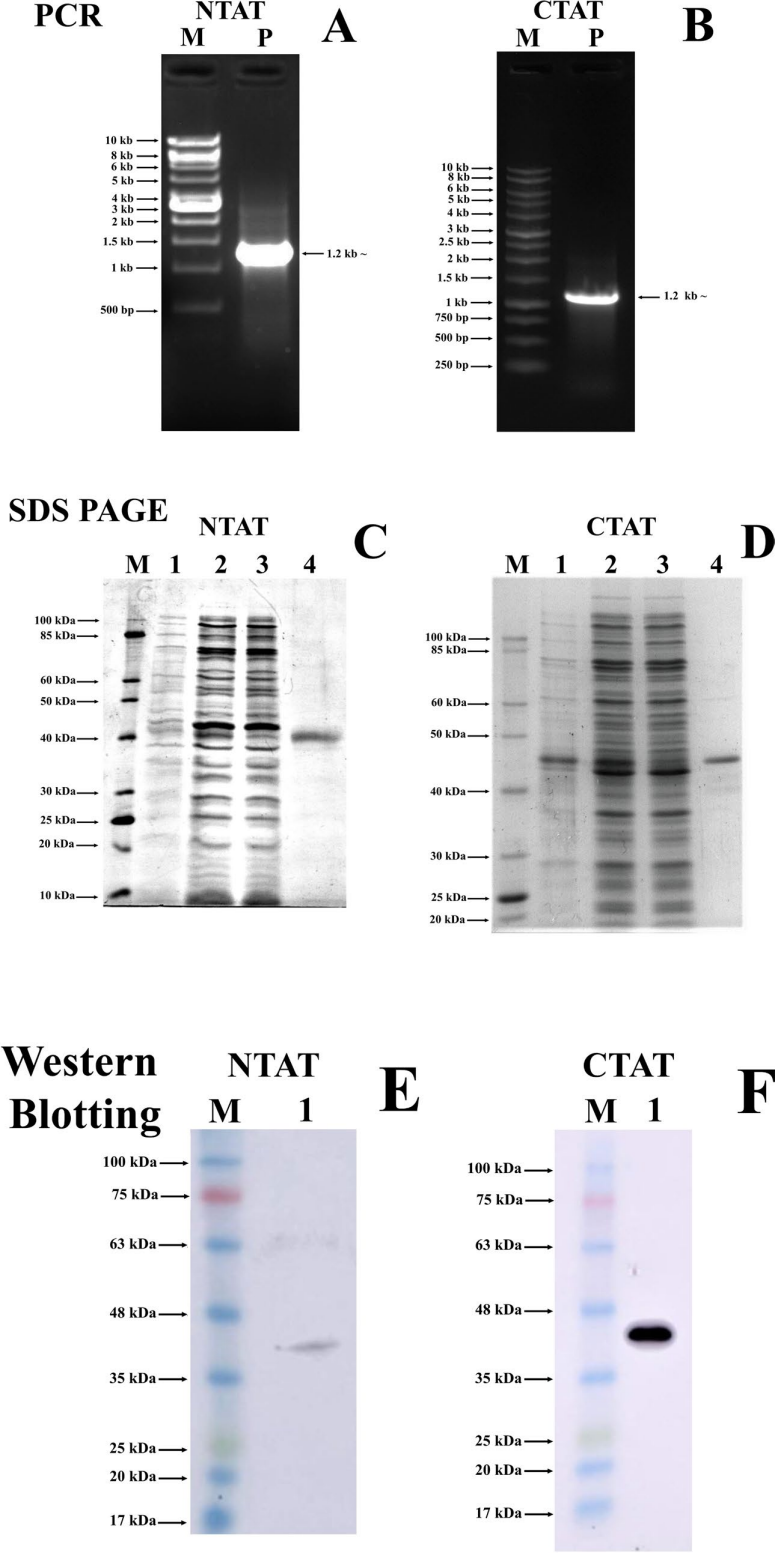
Cloning, purification and western blotting of NTAT and CTAT: (**A**) and (**B**) PCR amplified product of NTAT and CTAT exhibiting a band at 1.2 kb ~ respectively. Lane M: 1 kb ladder (NEB for NTAT and Promega for CTAT) Lane P: PCR product of NTAT and CTAT at 1.2 kb. (**C**) and (**D**) 12% SDS PAGE analysis displaying purified NTAT and CTAT respectively. Lane M: 10–100 kDa protein ladder (NEB) Lane 1: Un-induced NTAT and CTAT BL21 cells respectively. Lane 2: Sonicated supernatant showing NTAT and CTAT BL21 induction with 0.5 mM IPTG at 20 °C overnight, respectively. Lane 3: Flow-through after Ni–NTA binding Lane 4: Purified protein of NTAT and CTAT indicating expression at 43 kDa and 45 kDa respectively. (**E**) and (**F**) Western blot with anti-His antibody Lane M: 17–100 kDa pre-stained protein ladder (Abcam) Lane 1: Anti-His blots displaying luminescent bands between 35 and 48 kDa.

The PCR amplification of NTAT and CTAT exhibited a band with a size of approximately 1,167 bp (Fig. 3A,B). Positive clones from kanamycin-resistant plates were confirmed by colony PCR. The restriction digestion of the NTAT and CTAT from the pET28a(+) vector using BamHI and HindIII was also performed for confirmation (Supplementary Data 1). To confirm the sequence construct and frame of insert, the construct was sequenced and verified by comparing it with the TAT sequence from *Leishmania donovani* (GenBank id: MK426678) (Supplementary Data 2). Protein expression of NTAT and CTAT was optimized and the expression was confirmed by running on 12% SDS-PAGE gel. NTAT expressed a band of approximately 43 kDa and CTAT expressed a band of approximately 45 kDa due to the presence of two His-tags at both N and C-terminal (Fig. 3C,D). The size of the bands were estimated and confirmed by calculating the relative migration of the purified NTAT and CTAT bands in SDS-PAGE gel with the help of ImageJ software. Western blot performed with anti-His antibody further confirmed the presence of His-tagged NTAT and CTAT at their respective molecular weight of approximately 43 kDa and 45 kDa (Fig. 3E,F). All uncropped gel images are provided in Supplementary Data 5.

### Kinetic characterization of NTAT and CTAT

**Figure 4:**
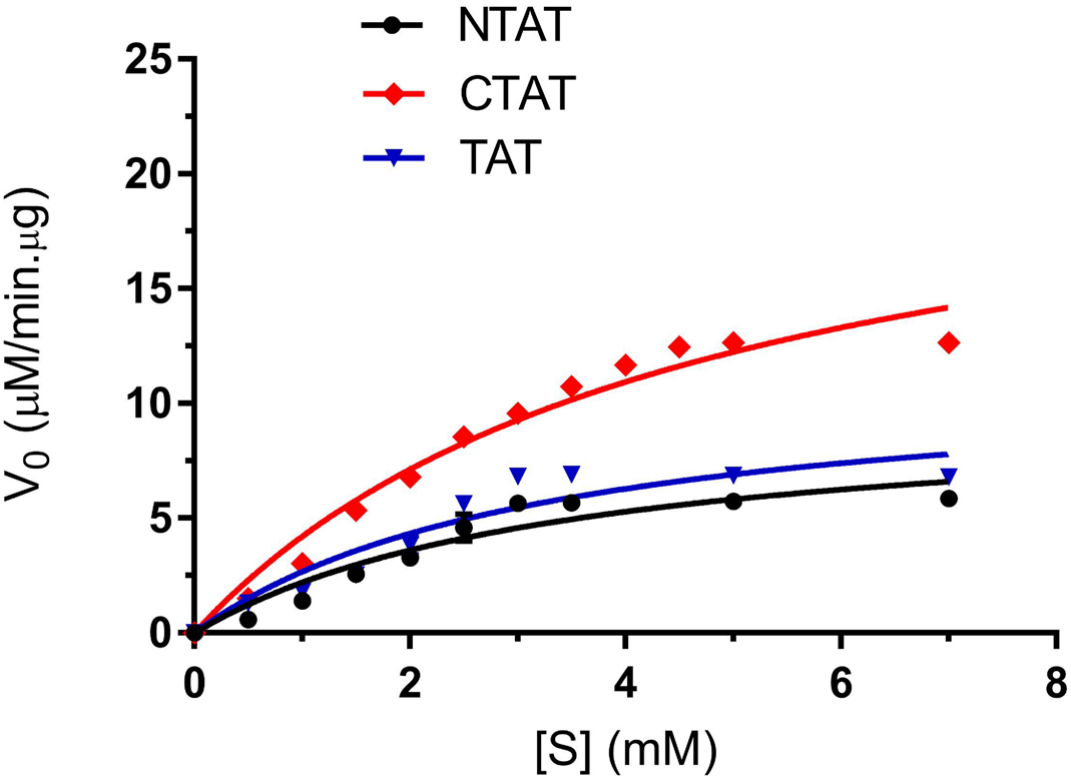
Michaelis–Menten Plot for NTAT, CTAT and TAT: The plot shows the activity of CTAT to be higher with respect to NTAT and TAT activity. The reduced activity of NTAT might be due to the lower binding of PLP to the active site cavity and residue K286. *K*_*m*_ of NTAT, CTAT and TAT were calculated to be 3.468 ± 0.796 mM, 4.581 ± 0.627 mM and 3.282 ± 0.799 mM respectively. All experiments were conducted in triplicates and SEM was calculated.

Kinetic characterization of NTAT and CTAT was conducted to compare the *K*_*m*_ and *V*_*max*_ of the truncated proteins with that of TAT from *Leishmania donovani*. When tyrosine and sodium pyruvate were used as the substrate and co-substrate, the *K*_*m*_ of NTAT and CTAT was found to be 3.468 ± 0.796 mM and 4.581 ± 0.627 mM respectively. In our previous study, TAT was found to have a *K*_*m*_ value of 3.282 ± 0.799 mM for the tyrosine-sodium pyruvate pair^9^. *V*_*max*_ of the truncated proteins was also analyzed and calculated to be 9.870 ± 1.132 µM/min µg for NTAT and 23.473 ± 1.748 µM/min µg for CTAT. Whereas, TAT had a *V*_*max*_ value of 11.451 ± 1.363 µM/min µg. The Michaelis–Menten plot for NTAT, CTAT, and TAT was calculated using GraphPad Prism 7 and shown in Fig. 4**.** The *K*_*m*_ of CTAT was higher than NTAT and TAT whereas the *V*_*max*_ was calculated to be higher for CTAT, lesser in TAT and much lower in NTAT. ANOVA analysis showed that CTAT exhibited a significant change in the activity when compared to TAT with a *P* < 0.05. The statistical analysis of NTAT’s activity with TAT was calculated to be insignificant with *P* > 0.05. Therefore, it was concluded that there is a significant increase in the activity of CTAT and an insignificant change in NTAT’s activity when compared to TAT.

### Effect of temperature and pH on truncated NTAT and CTAT

**Figure 5:**
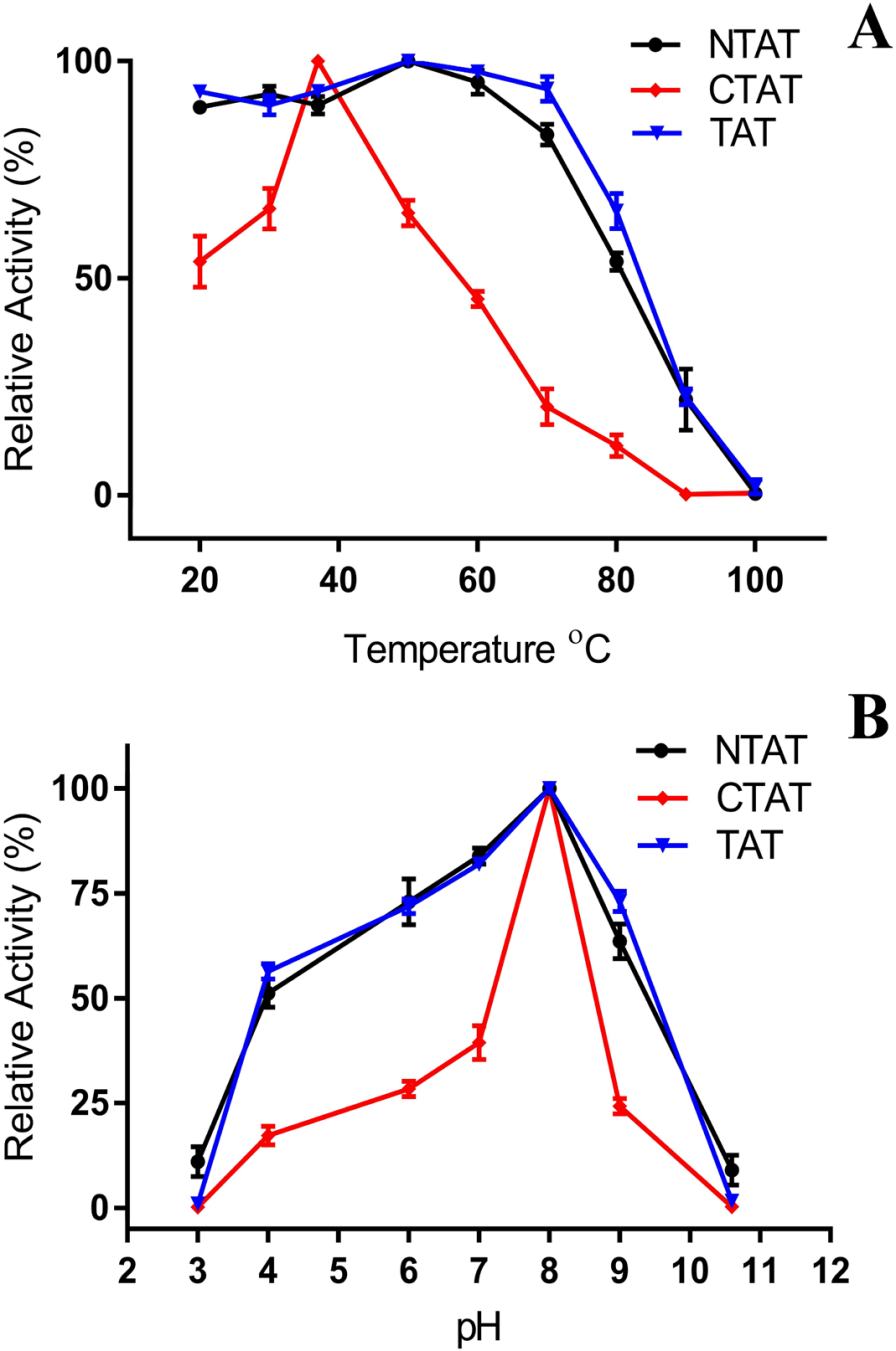
Activity of NTAT and CTAT at various pH and temperature: (**A**) The activities of NTAT and CTAT were carried out at various temperatures and their relative activities were calculated. NTAT displayed activity similar to that of TAT while CTAT was sensitive to temperature variation largely as seen from the plots. (**B**) The pH stability of NTAT and CTAT was checked at various pH and their activities were plotted in terms of relative activity. NTAT, as seen in temperature profile, remained stable relative to TAT^9^ but CTAT displayed reduced activity at extreme pH conditions.

The effect of temperature and pH on the truncated proteins were studied to understand the tolerance of NTAT and CTAT towards temperature and pH when compared to TAT. The effect of 60 amino acids truncation was profound in CTAT than in NTAT. The relative activity of NTAT and CTAT at different temperature i.e. from 20 to 100 °C was measured and the results are represented in Fig. 5A. NTAT displayed temperature stability, which was almost similar to that of TAT, but there was a 5% decrease in activity at all the temperatures. CTAT displayed its highest activity at 30–40 °C but the activity of CTAT decreased drastically at temperatures higher than 40 °C. The pH stability and activity of the truncated proteins also exhibited a similar result. NTAT’s activity at different pH from 3–11 was again similar to that of TAT and the highest activity was recorded at pH 8. Also, NTAT remained stable from a pH of 4–9 and similar pH stability was found in our previous studies in TAT^10^. CTAT, on the other hand, failed to perform equally when compared with NTAT and TAT. The activity was found to be drastically decreased when the pH was adjusted to the acidic or basic range as shown in Fig. 5B**.** Overall, CTAT presented much lower temperature and pH stability when compared to NTAT and TAT.

### Urea denaturation studies on CTAT and NTAT

**Figure 6:**
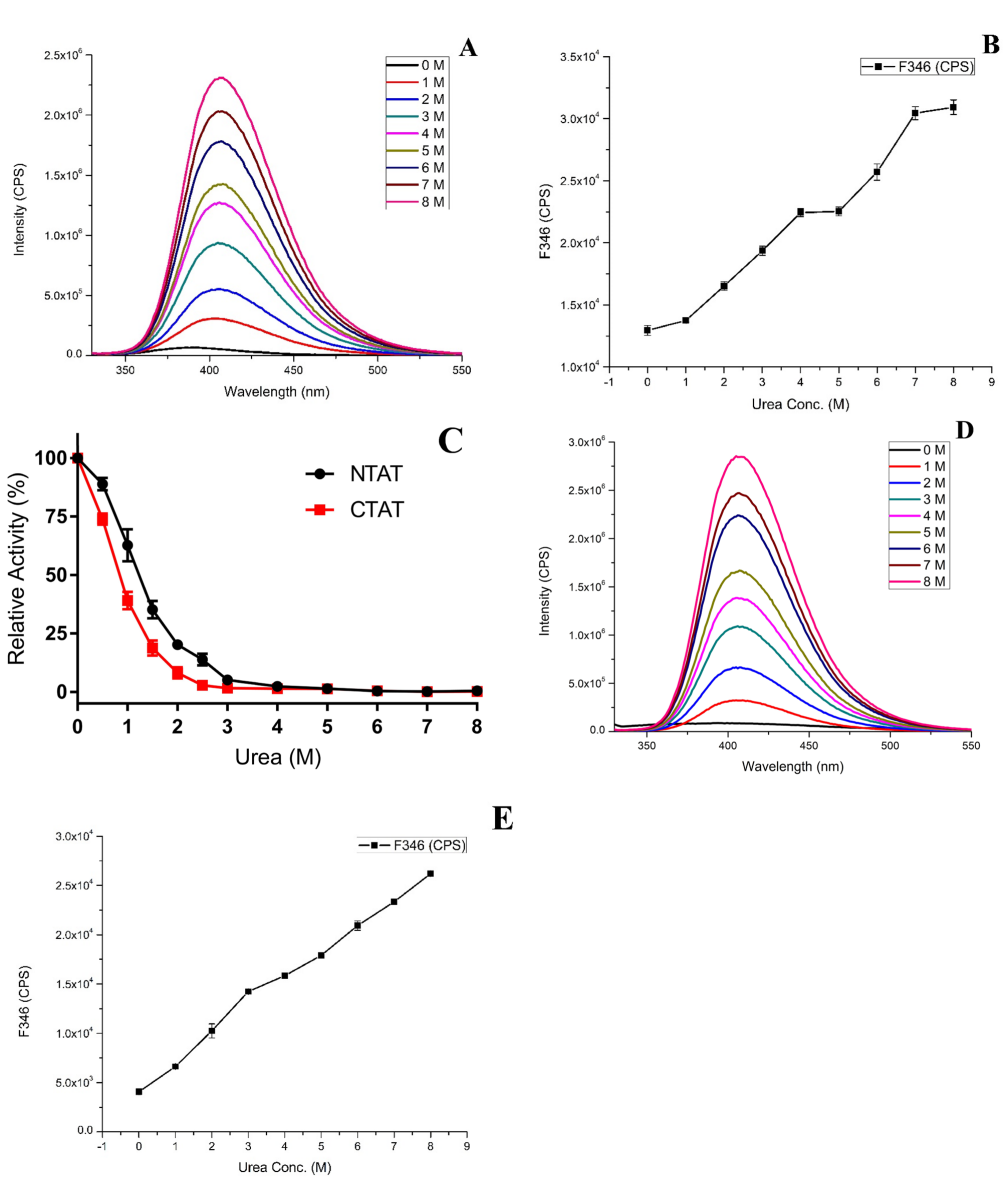
Urea denaturation and folding studies (**A**) and **(D)** Fluorescence intensity of NTAT and CTAT respectively measured at various urea concentrations. A steep increase in the intensity and redshift of emission maxima were observed in both the fluorescence plot. (**B**) and (**E**) The plot showing an increase in the fluorescence intensity of NTAT and CTAT respectively at 346 nm at increasing urea concentration. It is seen that NTAT exhibits N→X→D states with intermediate X state at 4–5 M urea while CTAT does not display any intermediate state of denaturation N→D (N-Native; D-Denatured). (**D**) Relative activity of NTAT (red) and CTAT (black) with increasing urea concentration was determined and NTAT showed activity till 2.5 M urea while CTAT became inactive at 2 M urea.

To determine the number of folding states and the activity of the proteins in the presence of urea denaturant, urea-based denaturation studies involving the calculation of relative activity and fluorescence intensity was carried out. NTAT activity in the presence of urea was recorded till 2.5 M urea, after which negligible activity was recorded till 8 M urea concentration. The intrinsically fluorescent amino acids like Trp, Tyr and Phe are present partially or fully buried inside the core of the folded protein or sometimes in the interface of the multi-domain protein. In NTAT, the fluorescent intensity of the protein increased with an increase in the concentration of urea (Fig. 6A). The increase in the fluorescence of LdTAT with respect to increased urea concentration was observed in our previous study^9^. The fluorescent intensity at 346 nm was analyzed and plotted as a function of urea concentration (Fig. 6B). There was a redshift observed in the fluorescence peaks with increasing urea concentration, indicating protein denaturation. The F346 analysis found the existence of an intermediate folding state (X) between 4 and 5 M urea concentrations. The completely denatured state of the NTAT protein was noticed at 7–8 M urea. In the case of CTAT, the activity of the protein gradually decreased till 2 M urea, and no further activity was detected from 3 M urea concentration (Fig. 6C). The fluorescence intensity of CTAT increased with respect to the increasing urea concentration as shown in Fig. 6D, and also the redshift in the fluorescence peaks was noticed. Interestingly, the intermediate state (X) was not observed in the F346 intensity analysis and a linear increase in the F346 intensity was found (Fig. 6E). TAT folding studies using urea denaturation from previous studies had shown the three-stage folding mechanism of N→X→D (N being native and D being denatured) wherein the intermediate state (X) was observed between 3 and 4 M urea concentrations with relative activity similar to NTAT. In the truncated proteins, NTAT exhibited N→X→D states with the intermediate state appearing between 4 and 5 M urea and CTAT displayed only N→D states. It is evident from the denaturation study that the C-terminal region undergoes a folding change during the enzymatic activity.

### PLP binding study using UV spectroscopy

**Figure 7:**
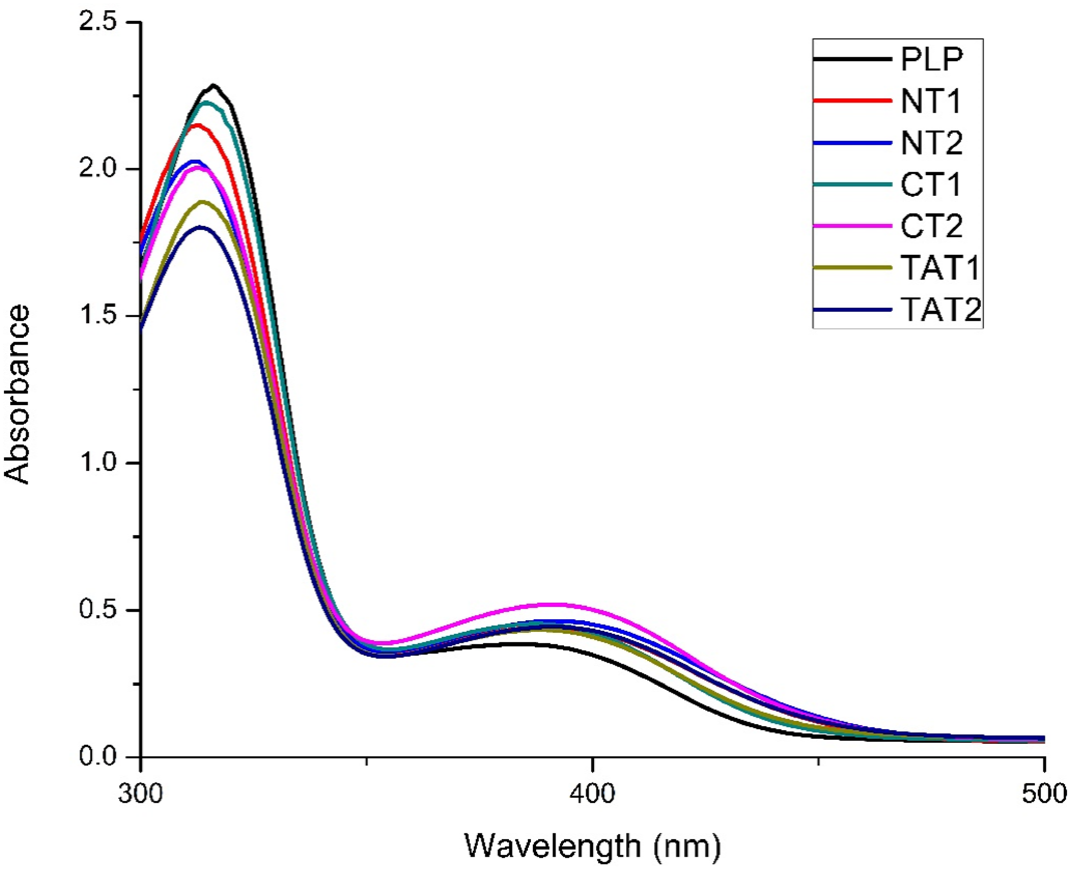
PLP binding studies: The absorption maxima of PLP at 320 nm and 420 nm was exploited to understand the formation of aldimine bonds. The formation of aldimine bond produces a blue shift in the peak at 320 nm and red shift at 420 nm along with an increase in intensity. Moreover, the binding of CTAT caused a high absorbance in the 420 nm peak maxima indicating better binding of PLP to K286. *PLP* Pyridoxal-l-phosphate, *NT1* PLP + 0.5 mg/ml of NTAT, *NT2* PLP + 1 mg/ml of NTAT, *CT1* PLP + 0.5 mg/ml of CTAT, *CT2* PLP + 1 mg/ml of CTAT, *TAT1* PLP + 0.5 mg/ml of TAT, *TAT2* PLP + 1 mg/ml of TAT.

UV absorption study for the detection of aldimine bond formation was conducted to understand the binding behavior of the co-factor PLP with the active site K286 of NTAT, CTAT, and TAT proteins. The successful binding and stabilization of PLP in the active site is revealed by a blue shift of 320 nm peak maxima and a redshift in the 420 nm peak maxima. A decrease in the absorbance of the 320 nm can also be detected along with an increase in the 420 nm absorbance due to the formation of aldimine GRbonds^13,14^. PLP bound much less to NTAT as observed from Fig. 7. The increase in NTAT concentration also showed a further decrease in the 320 nm intensity and a blue shift whereas, at 420 nm, the peak intensity rose higher. In CTAT, the PLP binding was higher than NTAT wherein the 320 nm peak absorbance was similar to NTAT but much higher absorbance was recorded in 420 nm peak intensity. The increase in the concentration of NTAT or CTAT bound more PLP and formed aldimine bonds thereby showing shifts in the UV absorption maxima. The study shows that PLP binding and stabilization required N-terminal more than the C-terminal and that the high binding of PLP to TAT as observed in Fig. 7 is an amalgamation of higher N-terminal stabilization and lesser C-terminal residue’s contribution.

### MD simulation of NTAT and CTAT

The energy minimized structures of NTAT and CTAT were initially checked for structural integrity using RAMPAGE and SAVES server. Ramachandran plot analysis had 94% and 89.8% of amino acids in the favored region and 4.7% and 8.1% of amino acids in the allowed region of NTAT and CTAT, respectively. SAVES server results also corroborated the quality of the NTAT and CTAT modeled structures. In SAVES, Verify 3D results of NTAT and CTAT displayed 94.85% amino acids and 95.94% of amino acids with a score ≥ 0.2. In Verify 3D, the structure is evaluated as “pass” if 80% of the residues are ≥ 0.2. ERRAT results of the SAVES server also measured an overall quality factor of 97.89 and 96.81 for NTAT and CTAT respectively out of 100. PLP molecule was then docked to these structures and the binding energy was calculated accordingly. PLP had binding energy of − 5.55 kcal/mol towards NTAT and − 5.71 kcal/mol towards CTAT. In NTAT docked PLP, amino acid residues S91, K226, Y196, and P231 formed hydrogen bonds with PLP initially whereas in CTAT, the PLP molecule formed hydrogen bonds with Y256, K286 and S151. The residues forming hydrogen bonds between TAT and PLP were found to be S151, Y256, K286 and P291 from our previous studies. It can be observed that PLP formed a hydrogen bond with K286 of CTAT but not of NTAT in the docking studies. The other free energies such as intermolecular energy, Van der Waals energy, electrostatic energy, total internal energy and torsional energy are shown in Table 1. The intermolecular energy, Van der Waals energy and electrostatic energy were higher for TAT when compared to NTAT and CTAT. Among CTAT and NTAT, CTAT had significantly higher intermolecular energy of − 7.8 kcal/mol compared to NTAT (− 7.64 kcal/mol). The electrostatic energy was also reduced in NTAT and CTAT when compared to TAT. This might be due to the truncation of several charged residues of the TAT structure. The other energies were similar for both NTAT and CTAT. The PLP bound structures of NTAT and CTAT are shown in Supplementary Data 3A,B.

**Table 1:** Binding free calculation of NTAT, CTAT and TAT obtained by docking PLP co-factor in Autodock v4.2.

Modeled Protein	Binding Energy (kcal/mol)	Intermolecular energy (kcal/mol)	Van der Waals energy (kcal/mol)	Electrostatic energy (kcal/mol)	Total internal energy (kcal/mol)	Torsional energy (kcal/mol)
CTAT	– 5.71	– 7.8	– 7.12	– 0.67	– 0.11	2.09
NTAT	– 5.55	– 7.64	– 7.0	– 0.64	– 0.12	2.09
TAT	– 6.26	– 8.35	– 7.53	– 0.82	– 0.09	2.09

**Figure 8:**
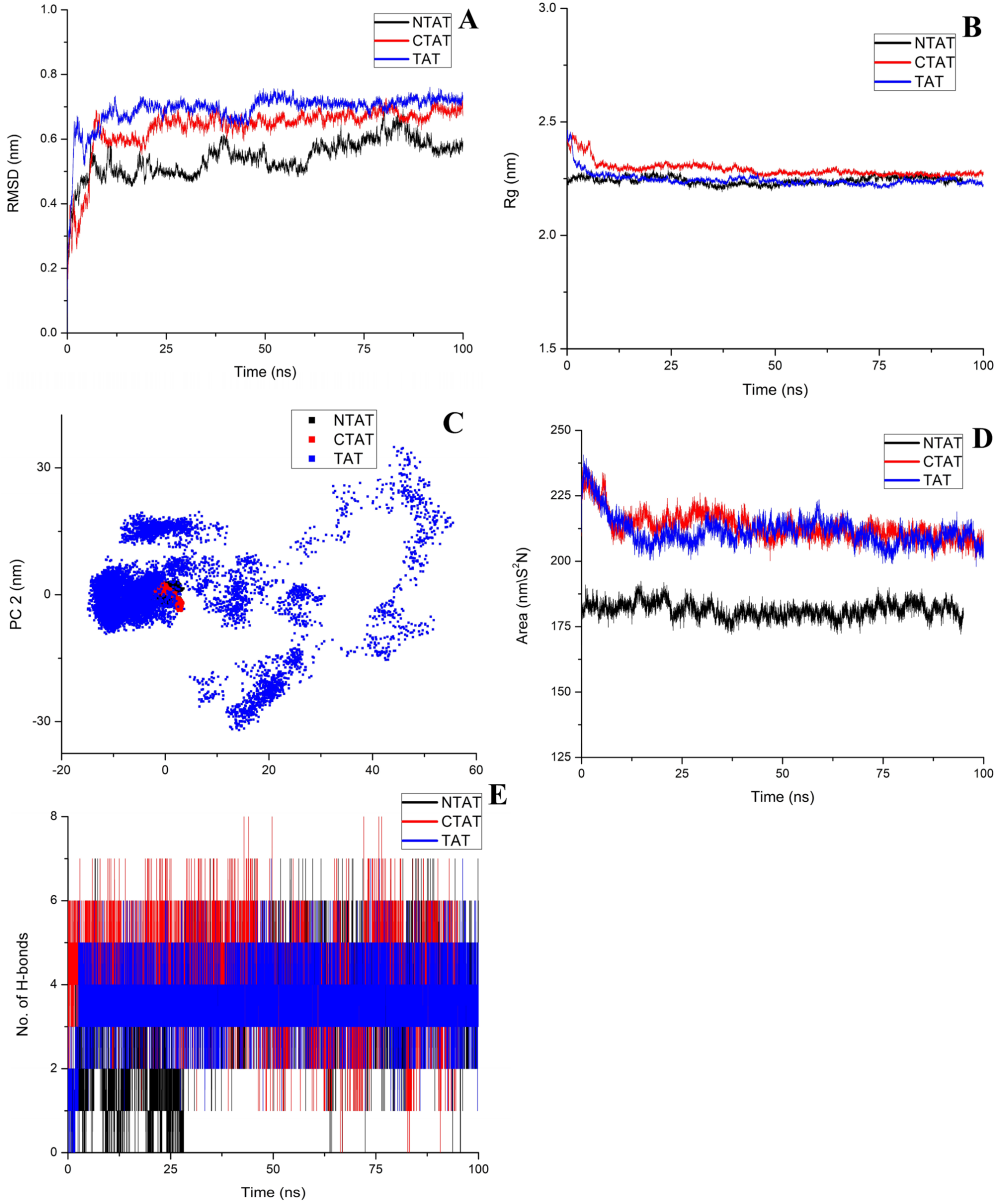
Trajectory analysis of molecular dynamic simulation: (**A**) RMSD analysis of NTAT (black), CTAT (red) and TAT (blue). The RMSD of NTAT was lower and stable than CTAT and TAT (Stability: NTAT > CTAT > TAT). (**B**) Radius of gyration of NTAT, CTAT and TAT. All the Rg analysis showed similar values thereby representing the compactness in the folding of the proteins. (**C**) The principal component analysis of NTAT, CTAT and TAT exhibited similar clustering and also the area covered by the vectors was also similar. This signifies that the structure remained relatively stable and rigid even after truncation. The binding of PLP did not produce any large scale motion in both the truncated proteins as well as TAT (**D**) SASA analysis of NTAT, CTAT and TAT found that the SASA of NTAT was very low when compared to the SASA values of TAT and CTAT. It was also observed that the SASA value of CTAT was higher than the TAT SASA value. (**E**) H-bond analysis of NTAT, CTAT and TAT with PLP is plotted in the figure. CTAT formed a higher number of H-bonds than TAT while NTAT had reduced H-bonding with PLP. The reduction in the formation of H-bonds might be due to the non-stabilization of co-factor PLP in the active site as perceived from the UV studies.

The bound structures were then simulated for 100 ns using the GROMACS package and the trajectories were analyzed. PLP bound with TAT was maintained as a reference in all the trajectory analysis. Root Mean Square Deviation (RMSD) of the backbone atoms for NTAT and CTAT was calculated and was found to be 0.54 ± 5e^−4^ nm, 0.6 ± 7e^−4^ nm, and 0.69 ± 5e^−4^ nm for NTAT, CTAT and TAT, respectively. RMSD of Cα atoms was calculated to be 0.25 ± 7e^−4^ nm, 0.64 ± 7e^−4^ and 0.68 ± 4e^−4^ for NTAT, CTAT and TAT, respectively (Supplementary Data 4A). RMSD plots of backbone and Cα atoms suggest a stable structure of the modeled proteins after 10 ns of MD run (Fig. 8A). RMSD-Cα plot suggests a well-folded and compact structure of NTAT when compared to CTAT and TAT. To understand the spatial spread of the protein’s mass, Radius of gyration (Rg) was measured. Rg analysis for the modeled proteins displayed extreme stability after 10 ns wherein the Rg values were measured to be 2.24 ± 1e^−4^, 2.29 ± 3e^−4^ and 2.24 ± 2e^−4^ for NTAT, CTAT and TAT respectively. It was noticed that the NTAT and TAT are packed compactly in a similar way, therefore, the activity difference was very meager when compared to the CTAT compact folding (Fig. 8B). Root Mean Square Fluctuations of the modeled protein were also analyzed to understand the fluctuations in specific regions. NTAT protein exhibited high fluctuations in the first few residues (T61, D62, N63, M64 and K65) near the truncated terminal region which might be due to the truncation and the unconnected coil structure of that region. Apart from that, NTAT showed very little fluctuation in the structure and the active site along with conserved regions around it were also rigid in nature. In CTAT, the N-terminal portion exhibited a higher fluctuation of ~ 0.75 nm. The other regions of the protein along with the active site was found to have very little fluctuations. Also the conserved amino acid residues in the C-terminal of NTAT had very few fluctuations similar to TAT (~ 0.1 nm). In TAT, the conserved regions of N-terminal had fluctuations similar to CTAT (0.4–0.1 nm) and other fluctuations observed were similar in NTAT and CTAT throughout the protein structure (Supplementary Data 4B).

The principal component analysis of NTAT, CTAT, and TAT was computed to decide the large scale motions that are essential in the functioning of the proteins. The eigenvalues and eigenvectors were obtained and the first two vectors representing the largest motions were plotted (Fig. 8C). It was corroborative evidence that the structure remained compact and without any large scale motions even after truncation. The PLP bound structures of NTAT and CTAT had motions very similar to the TAT and the vectors covered an area equal to that of TAT. To determine the surface area exposed to the solvent, the Solvent Accessible Surface Area (SASA) was computed from the trajectories as 181.3 ± 0.03 nm/S^2^N, 213.25 ± 0.05 nm/S^2^N and 211.55 ± 0.05 nm/S^2^N for NTAT, CTAT and TAT respectively. Surprisingly, it was seen that the SASA for NTAT was significantly less when compared to TAT and CTAT (Fig. 8D). This decreased SASA value might be the reason for the decreased NTAT activity observed before. In the CTAT protein, the SASA was slightly higher than that of TAT. The number of H-bonds formed between PLP and the truncated proteins and TAT was found to be 3.2 ± 0.01, 4 ± 0.01 and 3.5 ± 0.009 respectively. The number of H-bonds was found to be higher for CTAT when compared to NTAT and TAT as represented in Fig. 8E. This was in accordance with the SASA value where the solvent-accessible surface area was less for NTAT resulting in fewer hydrogen bonds with PLP. CTAT, which had higher SASA value, formed a higher number of hydrogen bonds and this was in corroboration with the activity analysis and UV spectroscopy studies. The in silico analysis concludes and supports the results that reduced activity of NTAT should be, due to the reduced SASA and the increase in CTAT activity might be due to the increased SASA of CTAT observed in the trajectory analysis.

## Discussion

Tyrosine aminotransferase is a broad aminotransferase that has been well-characterized in several organisms. The characterization of the enzyme TAT in *Leishmania donovani* was previously conducted in our lab but the extreme stability of the enzyme has not been attributed to any region of the structure before. The enzyme plays a major role in the metabolism of amino acids inside the parasite and this role is interwoven with various important pathways in the parasite. Two of the important pathways include polyamine pathway and salvage pathway that maintain the nucleotide and redox metabolism in the parasite. The enzyme is also assumed to be involved in the tocopherol synthesis pathway of the *Leishmania* parasite. Fumarate and acetoacetic acid, which are products in the pathway, are required for glucose metabolism and Kreb’s cycle. To decode the extreme stability of the enzyme and the function of a non-conserved N-terminal and conserved C-terminal, this study involved a cocktail of in vitro and in silico analysis.

NTAT, in which the N-terminal was truncated, was cloned into pET28a(+) vector and the protein was expressed and purified using N-NTA affinity chromatography. The N-terminal region of the TAT protein is very unique and has only 6 amino acids identical to the tyrosine aminotransferase of organisms like *Homo sapiens* and *Rattus norvegicus*. The His-tagged protein was purified and further confirmed by western blotting using an anti-His antibody. The kinetic characterization of NTAT using tyrosine-sodium pyruvate substrate-co-substrate pair gave a *K*_*m*_ value of 3.468 ± 0.796 mM and *V*_*max*_ of 9.870 ± 1.132 µM/min µg. The *K*_*m*_ value was similar to the TAT from *L. donovani* and the *V*_*max*_ value was 15% less when compared to TAT. ANOVA analysis concluded that the change in activity is insignificant (*P* > 0.05) when compared to TAT. In an earlier study, TAT from *Trypanosoma cruzi* exhibited a *K*_*m*_ value of 2.3 ± 0.2 mM^15^ in previous studies and the site-directed mutation of R238E did not change the *K*_*m*_ value (2.7 ± 0.3 mM) significantly^16^. A similar study involving TAT from *Trypanosoma cruzi* showed a *K*_*m*_ value of 6.4 ± 0.9 mM^17^. *Trypanosoma rangeli* TAT measured a specific activity of 0.284 U mg^−1^ in the induced bacterial lysate. Another study of TAT from *Leishmania major* had shown a *K*_*m*_ of 1.8 ± 0.1 mM and *V*_*max*_ of 78 ± 3 U mg^−1^. The temperature and pH relative activity curves were also significantly similar with maximum activity recorded at 50 °C and at pH 8. The protein was stable and functional in the presence of urea till 2.5 M urea concentration and exhibited N→X→D states of folding in the urea denaturation studies using fluorescence spectroscopy. The intermediate X state was observed between 4 and 5 M urea concentration, unlike the TAT intermediate X state that was found between 3 and 4 M urea. UV spectroscopic studies to determine the binding of PLP to the NTAT protein resulted in the conclusion that PLP binds much less to NTAT when compared to TAT, yet, the formation of aldimine bonds was observed at 420 nm. PLP docked with NTAT had binding energy of − 5.55 kcal/mol but interestingly, there were no H-bonds attached to K286 which might be another reason for the reduced activity of NTAT. To corroborate the *in-vitro* studies at the molecular level, molecular dynamics simulation of NTAT with PLP was conducted. NTAT bound with PLP showed an extremely stable structure but the SASA value was reduced which could be the reason why PLP binds much less to the NTAT protein as observed in PLP binding study. The number of H-bonds between PLP and NTAT was also reduced when compared to TAT. The results lead us to hypothesize that the N-terminal of TAT is very essential for the stabilization of the co-factor PLP in the active site. The deletion of N-terminal, results in lowered activity because of PLP’s instability in the active site cavity. There might be multiple amino acid residues responsible for the stabilization mechanism in the N-terminal as the N-terminal does not contain many conserved residues or domains.

The deletion of 60 amino acids in the C-terminal domain of TAT brought new insights about the functioning of the conserved residues of C-terminal. Multiple sequence alignment results showed that around 29 amino acid residues out of 60 are conserved across most of the organism’s tyrosine aminotransferase. This intrigued the authors to truncate this domain and understand the function of the conserved C-terminal portion. The CTAT was cloned into the pET28a(+) vector and the expressed His-tag protein was purified using Ni–NTA affinity chromatography. The purified protein was then confirmed by the anti-His antibody and the kinetic characterization of the CTAT protein was conducted. The purified CTAT protein exhibited a *K*_*m*_ of 4.581 ± 0.627 mM and *V*_*max*_ of 23.473 ± 1.748 µM/min µg. The activity was found to be very high when compared to the wild TAT from *L. donovani*. ANOVA analysis for statistical significance found a significant change in the activity of CTAT when compared to TAT (*P* < 0.05). The temperature and pH characterization found that CTAT was stable only at pH 8 and at 30–40 °C temperature range after which the activity decreased significantly. Previous studies on tyrosine aminotransferase from mice had also reported increased temperature and pH stability of the enzyme^11^. Intrinsic fluorescence studies employing urea denaturant found that the CTAT had no intermediate X state (N→D) exhibited by NTAT and TAT and the F346 intensity increased linearly with an increase in urea concentration. The disappearance of the intermediate X state upon deletion of the C-terminal was interesting as it deviated from the N→X→D states observed in NTAT and TAT. The UV spectroscopic studies found a higher binding of PLP when compared to NTAT. Shifts were also observed on the addition of enzyme to the PLP molecule. The increased peak at 420 nm for CTAT showed higher aldimine bond formation. In silico docking studies with PLP had binding energy of − 5.71 kcal/mol and the docked structure was further simulated to find out the interactions at the molecular level. CTAT docking with PLP formed hydrogen bonds with amino acid residues similar to that of TAT docked PLP, moreover, hydrogen bond with K286 of CTAT was observed clearly. The CTAT bound PLP structure was stable as analyzed from RMSD, RMSF and Rg analysis. SASA analysis showed a higher surface area exposure to the solvent when compared to the TAT and NTAT structure. This evidence supports the higher PLP binding to CTAT observed in the UV spectroscopy studies. On calculating the H-bonds formed, the number of H-bonds was higher than NTAT and TAT. The activity of the protein was, therefore, higher as the PLP molecule was stabilized in the active site cavity with the help of the N-terminal domain. But the exposure of CTAT to extreme conditions reduced the activity. The authors hypothesize that the intermediate state observed in NTAT and TAT might be the unfolding of the C-terminal that protects the active site cavity with the help of charged residues in the C-terminal. Since the intermediate state was not observed in the CTAT protein and the susceptibility of CTAT to temperature and pH increased, the authors believe that the amino acids in the C-terminal conserved region might be responsible for the increased protein stability towards temperature and pH.

This study of systematically deleting the N-terminal and C-terminal domain was carried out at both in vitro and in silico levels and mapping of the functions was performed accordingly. The functional characterization of NTAT and CTAT helped us understand that N-terminal stabilizes the co-factor PLP in the active site cavity and that the C-terminal confers tolerance against extreme pH and temperature conditions. The biphasic folding mechanism of NTAT and the single-phase folding mechanism of CTAT along with PLP binding studies corroborated the functions attributed to the C- and N-terminal domains. Furthermore, the study revealed the function of the much conserved C-terminal domain of TAT in various organisms. Concluding the study, TAT from *L. donovani* has a non-conserved N-terminal and a conserved C-terminal whose functions have been mapped by gene truncation strategy. The reason for the high stability of the protein at extreme conditions is a question that still remains unanswered. From the unique function and sequence of the enzyme, it is evident that the enzyme does play a major role in the metabolism and survival of the parasites.

## Materials and methods

### Sequence analysis

Multiple sequence alignment was conducted to understand the conserved and non-conserved amino acids present in the N- and C-terminals of tyrosine aminotransferase. The analysis of the tyrosine aminotransferase from different organisms was done using sequences obtained from the GenBank database^19^. The sequences were then subjected to multiple sequence alignment using Clustal Omega^20^. TAT from *L. donovani* was compared with NTAT, CTAT and TAT sequences from a prokaryote i.e. *E. coli* (in which LdTAT, NTAT, and CTAT proteins are being expressed), *Rattus norvegicus* and *Homo sapiens* (host organisms for *Leishmania parasite*) and *Trypanosoma cruzi* (which is a close relative of *L. donovani* parasite). The TAT sequence of *Homo sapiens* was also chosen for multiple sequence alignment to demonstrate the capacity of TAT from *Leishmania donovani* as a drug target. The output format in Clustal Omega was set as ClustalW with character counts and all other parameters in Clustal Omega were set as default for multiple sequence alignment. The sequences were then represented using ESPript 3.0^21^. The identical sequence regions are represented in highlighted red with white characters and the gaps are shown in dotted lines in the figure.

### Cloning, expression, and purification of NTAT and CTAT

NTAT and CTAT of size 1,167 bp were amplified using genomic DNA from *Leishmania donovani.* The complete tyrosine aminotransferase sequence of *Leishmania donovani* is 1,347 bp (GenBank id: MK426678) and 180 bp was truncated from N-terminal in NTAT and C-terminal in CTAT. A schematic representation of the truncation of N-terminal and C-terminal domains are provided in Fig. 1. The gene-specific primers for the amplification of NTAT and CTAT were conducted using primers as listed in Table 2. All chemicals used in this study were procured from Sigma-Aldrich (St. Louis, MO, USA) and Merck K GaA (Darmstadt, Germany) unless otherwise specified. The amplified products and pET28a(+) vector were sequentially digested using BamHI (NEB) and HindIII (NEB) restriction enzymes and the ligation was conducted overnight at 4 °C using T4 DNA ligase (NEB). The ligated samples were transformed into *E. coli* (DH5α strain). Selection of positive clones was performed by incubating the kanamycin LB-agar plates at 37 °C. The positive clones were picked and further confirmed using colony PCR and restriction digestion. The isolation of plasmids from the positive colonies was conducted manually and transformed into *E. coli* (BL21 strain). Following this, protein expression was performed by growing the NTAT and CTAT BL21 strains to 0.6 OD. The induction of both the His-tagged proteins was carried out by using 0.5 mM of IPTG at 20 °C overnight. The cells were then harvested by centrifugation and sonicated in lysis buffer (20 mM Tris HCl, 500 mM NaCl, and 50 mM imidazole) with 50 pulses of 5 s on/10 s off cycle. After incubating the sonicated samples in ice for another 30 min, they were centrifuged at 9,500 rpm, 30 min at 4 °C and the supernatants were carefully collected. The Ni–NTA column was equilibrated with 3 column volumes of EQ buffer (20 mM Tris HCl, 200 mM NaCl and 20 mM imidazole). Following equilibration, the collected supernatants (15 mg/ml) were incubated in the Ni–NTA column for 45 min at 4 °C. The affinity column was then washed with 10 column volumes of Wash buffer I (20 mM Tris HCl, 500 mM NaCl and 50 mM imidazole) and five column volumes of Wash buffer II (20 mM Tris HCl and 500 mM NaCl). The His-tagged NTAT and CTAT proteins were subsequently eluted by adding 1 ml of elution buffer (20 mM Tris HCl, 500 mM NaCl and 500 mM imidazole) sequentially. The purified protein was obtained in the first 3 ml and no further elution was observed. The dialysis of the NTAT and CTAT proteins was performed and the final concentration of the buffer was brought down to 10 mM Tris HCl and 200 mM NaCl. A 12% SDS- PAGE gel was run to confirm the presence of purified protein (2.5 µg/ µl). The concentration of the purified protein was then estimated using Bradford’s assay^22^. TAT from *Leishmania donovani* which was previously cloned and purified in our lab^9^, was maintained as a reference in all the studies. NTAT and CTAT (2.5 µg/ µl) were blotted onto polyvinylidene fluoride (PVDF) membrane and after transfer, subsequently, blocking was carried out using 5% skimmed milk. The membrane was washed using TBST (Tris-buffered saline: Tween-20) and then incubated with an anti-His primary antibody (1:5,000) overnight at 4 °C. Secondary horseradish peroxidase antibody (1: 10,000) was added and the bands were detected using Chemidoc by development with chemiluminescent HRP substrate^23^.

**Table 2:** Gene-specific primers: Primers synthesized and used for the amplification of NTAT and CTAT.

S. no	Primer name	Primer sequence
1	NTAT-FP	CGC**GGATCC**ACCGACAACATGAAGCCT
2	CTAT-FP	ATATTA**GGATCC**ATGACGATTGATACGCAG
3	NTAT-RP	GATTTC**AAGCTT**CTACTTCTTGTGGCGCTC
4	CTAT-RP	GATTTC**AAGCTT**CTGAAAGTAGCTGAGGTT

The restriction sites utilized for cloning of the genes are marked in bold and underlined.

### Kinetic characterization of N-TAT and C-TAT

The activity of NTAT and CTAT was performed using tyrosine as substrate and sodium pyruvate as co-substrate according to Diamondstone, T.I, 1966^24^. The assay involved the incubation of NTAT and CTAT with tyrosine (0–7 mM) and 30 µM of pyridoxal 5′-phosphate (PLP) for 1 h at 37 °C, pH 8. Following this, sodium pyruvate of 100 mM was added and incubated for 10 min at 37 °C. The reaction was finally terminated by the addition of 10 N NaOH and the absorbance of the sample was measured at 331 nm after a period of 30 min using Shimadzu UV-1800 (UV–Vis spectrophotometer). 50 µg of the enzyme was employed for all the reaction assays performed. All the experiments were conducted in triplicates and the mean and SEM were calculated accordingly. ANOVA analysis was performed using GraphPad Prism 7 to understand the statistical significance between the two truncated enzyme’s activity with the wild type. The confidence interval of 95% was considered for statistical analysis.

### Effect of temperature and pH on NTAT and CTAT

To study the effect of temperature and pH on the stability on NTAT and CTAT, the enzymes were assayed at a temperature range from 20 to 100 °C and a pH range of 2–11. The assays were conducted at a constant substrate and co-substrate concentration of 4 mM tyrosine and 100 mM sodium pyruvate. All experiments were performed in triplicates and 50 µg enzymes were used for the study. The pH of the assay was varied by the use of different buffers like citrate buffer (pH 3 and 4), phosphate buffer (pH 6), HEPES buffer (pH 7), Tris (pH 8), MOPS buffer (pH 9) and sodium carbonate buffer (pH 11). The temperature studies were performed by the incubation of the enzyme at the desired temperature during the co-substrate incubation period of 10 min.

### Folding mechanism of NTAT and CTAT by urea denaturation:

NTAT has 12 Tyr and 6 Trp residues and CTAT has 11 Tyr and 6 Trp residues in the sequence and structure of the protein. The property of intrinsic fluorescence of the enzymes was tapped to analyze the structural changes at various conditions in the experiment. The folding mechanism of NTAT and CTAT was studied using urea as a chaotropic agent in this study. The activity of NTAT and CTAT was measured at urea concentrations from 0–8 M. The relative activity of the enzyme was also calculated according to the assay mentioned earlier and plotted accordingly. The fluorescence study was performed using Horiba Jobin–Yvon Fluorolog-3 (Model FL 3–21) equipped with a 1 cm quartz cell. The excitation and emission slits were set at 2 nm bandwidth for all the experiments at 25 ± 1 °C. 0.4 mg/ml of NTAT and CTAT was used for the fluorescence studies. The excitation wavelength of 295 nm was chosen and the emission spectrum was recorded between 340–550 nm. All scans were triplicated and mean was calculated followed by plotting. The F346 intensity against the concentration of urea was plotted for NTAT and CTAT to determine the folding states (N, X and D) in the enzymes.

### PLP binding study to NTAT and CTAT

The co-factor that binds to the TAT is PLP which binds to the anchoring site i.e. K286. The enzyme activity is primarily dependent on the attachment of the co-factor. The compound PLP displays two absorption maxima at 320 nm and 415 nm^13,14^. On binding of the enzyme to the PLP, there is a blue shift of 320 nm peak and red-shift of 415 nm absorption maxima. This is primarily due to the formation of the aldimine bond between PLP and K286. The UV-spectroscopy study was conducted by a spectrum scan from 300–500 nm. The experiments were conducted at a constant temperature of 25 °C and a 30 min incubation period was maintained after the addition of enzymes. The concentration of PLP was maintained at a concentration of 4 mM and two different concentrations of NTAT and CTAT 0.5 mg/ml and 1 mg/ml were chosen. A spectral scan of PLP was carried out followed by the addition of increasing concentration of the enzymes. All spectral scans were performed in triplicates and the average of three reads was plotted and analyzed.

### Modeling, docking and dynamic simulation of NTAT and CTAT

The structure of TAT from *Leishmania donovani,* previously modeled in our lab, was obtained from PMDB with PMDB id: PM0081305. The structure of TAT was modified by deleting 60 amino acids at the N-terminal and C-terminal of PM0081305 to achieve NTAT and CTAT models, respectively. UCSF Chimera was employed for the in silico deletion of PM0081305^25^. Energy minimization by steepest descent was conducted for NTAT and CTAT using GROMACS package v5.1.4. The energy minimized structures of CTAT and NTAT was then structurally validated using the SAVES server and RAMPAGE server^26,27^. The structures were further taken up for docking and simulation using Autodock v4.2^28^ and GROMACS package v5.1.4^29^. The energy minimized protein structure of NTAT and CTAT was assigned with Kollman charge and the ligand molecule was assigned with Gasteiger charge for docking studies. The ligand in this study was the co-factor PLP and therefore, the grid box was set around the K286 active site at 54 × 64 × 64 points. The electrostatic and desolvation maps were then generated using auto grid command and the final dock using NTAT and CTAT was performed for 500 runs of the Lamarckian-Genetic algorithm independently. For dynamic simulations, the ligand topologies were generated using ACPYPE^30^ and the topologies were manually screened for errors. The simulation of NTAT-PLP and CTAT-PLP for 100 ns involved the solvation of the system with TIP4 (Transferable Intermolecular Potential with 4 Points) water molecules in a dodecahedron box. The distance of the surface of the protein to the box edges was maintained at 10 Å. The neutralization of the system was carried out by the substitution of water molecules with 8 Na^+^ ions. Energy minimization was performed by the steepest descent integrator. After temperature and pressure equilibration of the system for 5 ns using Modified Berendsen thermostat (at 310 K) and Parrinello-Rahman barostat ( at 1 bar) respectively, the production MD run was carried out for 100 ns. The H-bond analysis between PM0081305 and PLP was obtained from our previous study^9^. The trajectories of the production run of NTAT, CTAT and TAT were analyzed using Xmgrace and the UCSF Chimera.

## Supplementary information

Supplementary Information.
