# Supplementary material for: Mapping N- and C-terminals of Leishmania donovani tyrosine aminotransferase by gene truncation strategy: a functional study using in vitro and in silico approaches
Source: Sci Rep. 2020 Jul 27;10:12463. doi: 10.1038/s41598-020-69512-y (PMC7385629; doi:10.1038/s41598-020-69512-y)
Supplement: Supplementary file 1 — Supplementary Information. [file 41598_2020_69512_MOESM1_ESM.pdf]

Mapping N- and C-terminals of *Leishmania donovani* tyrosine aminotransferase by gene truncation strategy: A functional study using in vitro and in silico approaches.

Santanu Sasidharan and Prakash Saudagar*

Department of Biotechnology, National Institute of Technology, Warangal-506004, Telangana, India

*Corresponding author

Dr. Prakash Saudagar

Department of Biotechnology,

National Institute of Technology, Warangal-506004, T.S.

E-mail: ps@nitw.ac.in

Phone no: +91 870 2462898

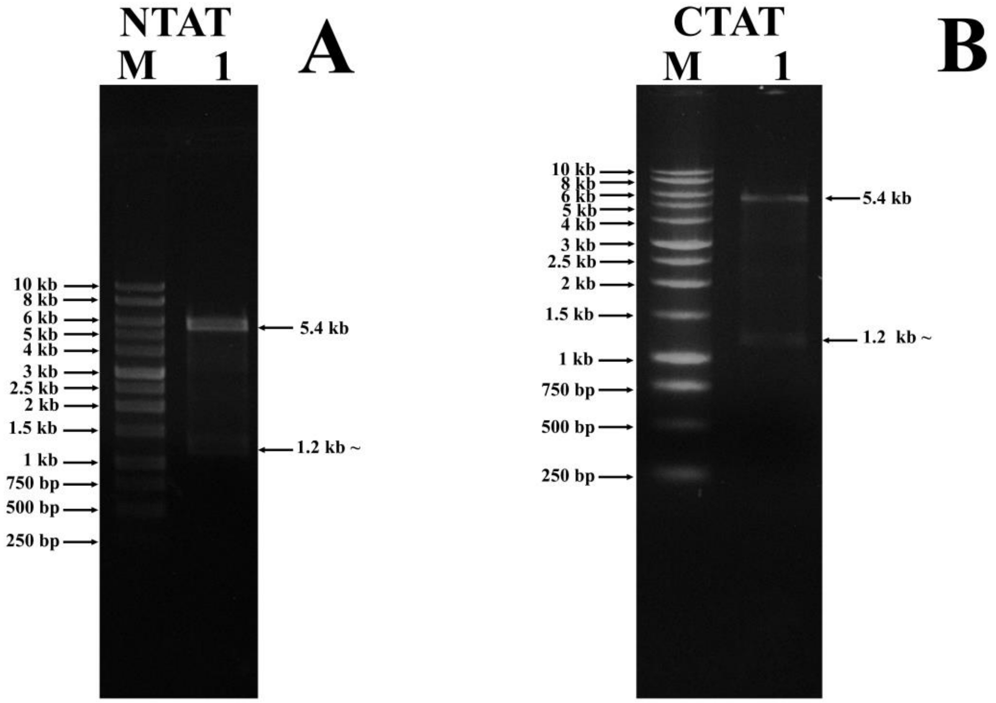

Supplementary data 1: Clone confirmation of NTAT and CTAT with BamHI and HindIII restriction digestion: (A) & (B) Lane M: 1kb DNA ladder (Promega) **Lane 1:** pET28a (+) vector band at 5.4 kb with the release of the insert at 1.2 kb ~ of NTAT and CTAT.

NTAT:

ACCGACAACATGAAGCCTTCCCGCTCCACTAAGTCGAACTTGCGTCTCTCCATCGGCGACCC
CACTGTCGACGGCAATCTAAAGACCCCCGACATTGTAACAGAGGCAATGGTAGATGTAGTG
CGCTCTGGCAAGTTCAACGGCTACCCGCCGACGGTCGGGGCAGACAACTTGCGCCAGGTGG
TGTCGACCTACTGGCGCCGCTTCTGCCAGACCAAGTCTCGTCAAGAGGCGTTGAAGTGGGAG
AACGTGATCATCACGTCCGGTGTGTCGCAGGCCATCGTGCTCGCTCTCACGGCACTGTGCAA
CGAGGGCGACAACATCCTCGTGTGCGCCCCATCGTTTCCCCACTACAAGAGCGTCTGCGACA
GCTACGGCATCGAGTGCCGCTACTATTACCTCGACCCCTCGAAGAGCTGGGAGTGCGACCTC
AGAGCTGCGGCCGGTATGGTGGATAGCCACACCAAGGCATTTGTCATCATCAACCCCTCCAA
CCCGTGCGGCAGCAACTTCTCCCGTGCACACGTGAGCGATATCATCGATTTCTGCCAGCAGC
ACCAGATCCCACTCATCAGTGACGAAATCTACGCTGAGATGGTGCTGAACAACGGCATCTTC
ACGTCTGTCGCCGACTTCGACACGAACGTTCCGCGTCTCATCCTGGGCGGCACAGCCAAGTA
TCAGGTCTGCCCCGGCTGGCGCGTAGGCTGGTCTATTCTGATCGACCCAATGAACGTTGCGG
GAGACTGGGCTGTCGGAATGGAGCGACTGACCCAGCTCATCGCTGGCGTCAACTCTATCTGC
CAGGAGGCGATTGCGCGGACACTGCTCAAGTGCCCGGCGGAGTGCACCGAGCACATCGTCA
CTCAACTGGAGGCCGGCGCCAAAGTGTACGCCCGACTGCTCGAACACGACATTGGCATCTCC
ATGGAGGCGCCGCAGGCCTCCATGTTCGTGATGCTCAAGCTGAACCTCAGCTACTTTCAGGA
TTTGAAGTCGGACATGGAGTTCTACGAGAAACTGCTGGATGAGGAGAACGTGCAGGTGCTG
CCGGGTGAGATCTTTGGCATGAGTGGCTTCCTTCGTGCAACGGTTTCCCGCCCATCGGCGGT
GCTGAATGAGGCAGTCGACCGCATCATCGAGTTCTGCGAGCGCCACAAGAAGTAG

CTAT:

ATGACGATTGATACGCAGGCCGCACCCGCTGCTCAAACCTTGAAGGCCGGCAACGCCACAG
GATCTCTCACCGAGGAGCGCCGCGAGGTCGAGCAGCAGCGCGCAGCTGAGAACACGAGTTT
TCGCCGTATCGCGTCCTCGAAGCACGCCCAGCGAACCCTGCAGCCGTTGAACAACTTGACCG
ACAACATGAAGCCTTCCCGCTCCACTAAGTCGAACTTGCGTCTCTCCATCGGCGACCCCACT
GTCGACGGCAATCTAAAGACCCCCGACATTGTAACAGAGGCAATGGTAGATGTAGTGCGCT
CTGGCAAGTTCAACGGCTACCCGCCGACGGTCGGGGCAGACAACTTGCGCCAGGTGGTGTC
GACCTACTGGCGCCGCTTCTGCCAGACCAAGTCTCGTCAAGAGGCGTTGAAGTGGGAGAAC
GTGATCATCACGTCCGGTGTGTCGCAGGCCATCGTGCTCGCTCTCACGGCACTGTGCAACGA
GGGCGACAACATCCTCGTGTGCGCCCCATCGTTTCCCCACTACAAGAGCGTCTGCGACAGCT
ACGGCATCGAGTGCCGCTACTATTACCTCGACCCCTCGAAGAGCTGGGAGTGCGACCTCAGA
GCTGCGGCCGGTATGGTGGATAGCCACACCAAGGCATTTGTCATCATCAACCCCTCCAACCC
GTGCGGCAGCAACTTCTCCCGTGCACACGTGAGCGATATCATCGATTTCTGCCAGCAGCACC
AGATCCCACTCATCAGTGACGAAATCTACGCTGAGATGGTGCTGAACAACGGCATCTTCACG
TCTGTCGCCGACTTCGACACGAACGTTCCGCGTCTCATCCTGGGCGGCACAGCCAAGTATCA
GGTCTGCCCCGGCTGGCGCGTAGGCTGGTCTATTCTGATCGACCCAATGAACGTTGCGGGAG
ACTGGGCTGTCGGAATGGAGCGACTGACCCAGCTCATCGCTGGCGTCAACTCTATCTGCCAG
GAGGCGATTGCGCGGACACTGCTCAAGTGCCCGGCGGAGTGCACCGAGCACATCGTCACTC
AACTGGAGGCCGGCGCCAAAGTGTACGCCCGACTGCTCGAACACGACATTGGCATCTCCAT
GGAGGCGCCGCAGGCCTCCATGTTCGTGATGCTCAAGCTGAACCTCAGCTACTTTCAG

Supplementary data 2: Nucleotide sequence data of NTAT and CTAT.

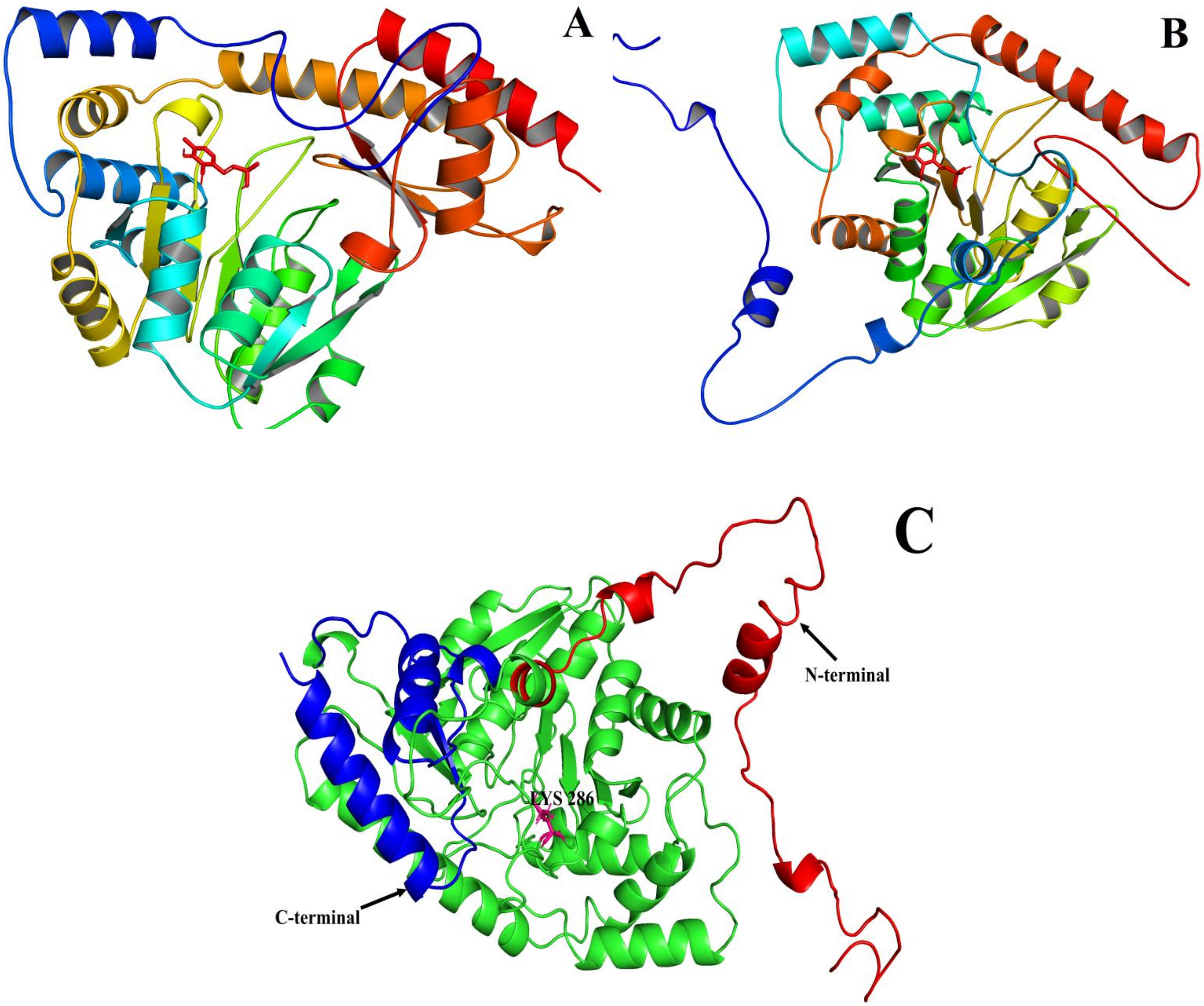

Supplementary Data 3: Docked pose of NTAT and CTAT with PLP: (A) The figure shows the co-factor PLP (in red) docked to NTAT in the active site of the enzyme. **(B)** The figure shows the C-terminal truncated CTAT with PLP (in red) docked in the active site cavity **(C)** Structure of complete tyrosine aminotransferase TAT with N-terminal in red and C-terminal in blue. The active site K286 is labeled and represented in magenta.

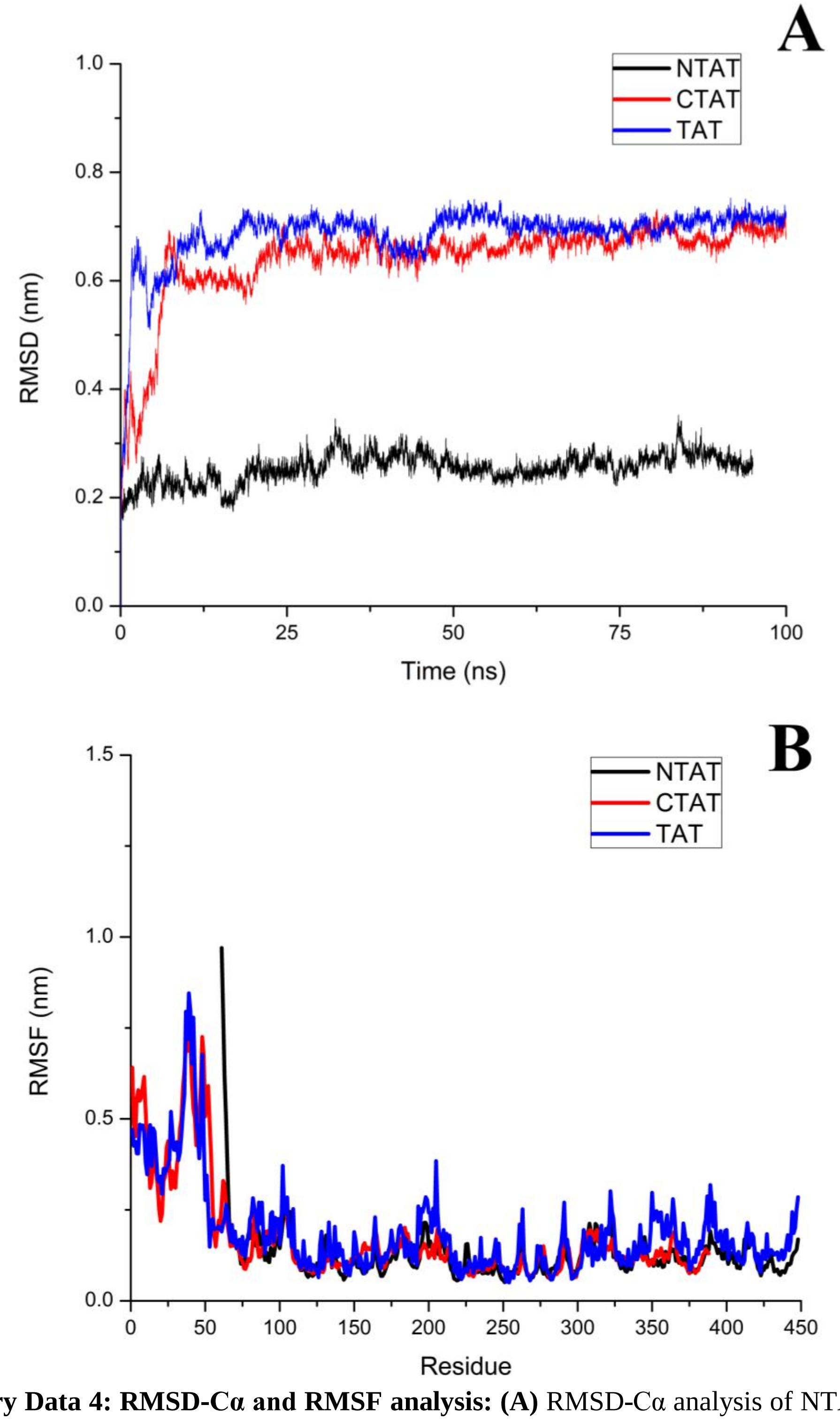

Supplementary Data 4: RMSD-Cα and RMSF analysis: (A) RMSD-Cα analysis of NTAT, CTAT and TAT is plotted in the figure. From the RMSD-Cα values, NTAT structure was found to be very stable when compared to CTAT and TAT. **(B)** The plot shows the local fluctuations present in the truncated NTAT and CTAT as well as TAT protein. The NTAT RMSF analysis shows fluctuations in few residues near the 60th – 70th amino acids. Other regions exhibited very little fluctuations. The active site residues were found to have fewer fluctuations in all the proteins considered and the active site cavity was found to be very rigid. CTAT also had fluctuations only at the N-terminal like TAT.

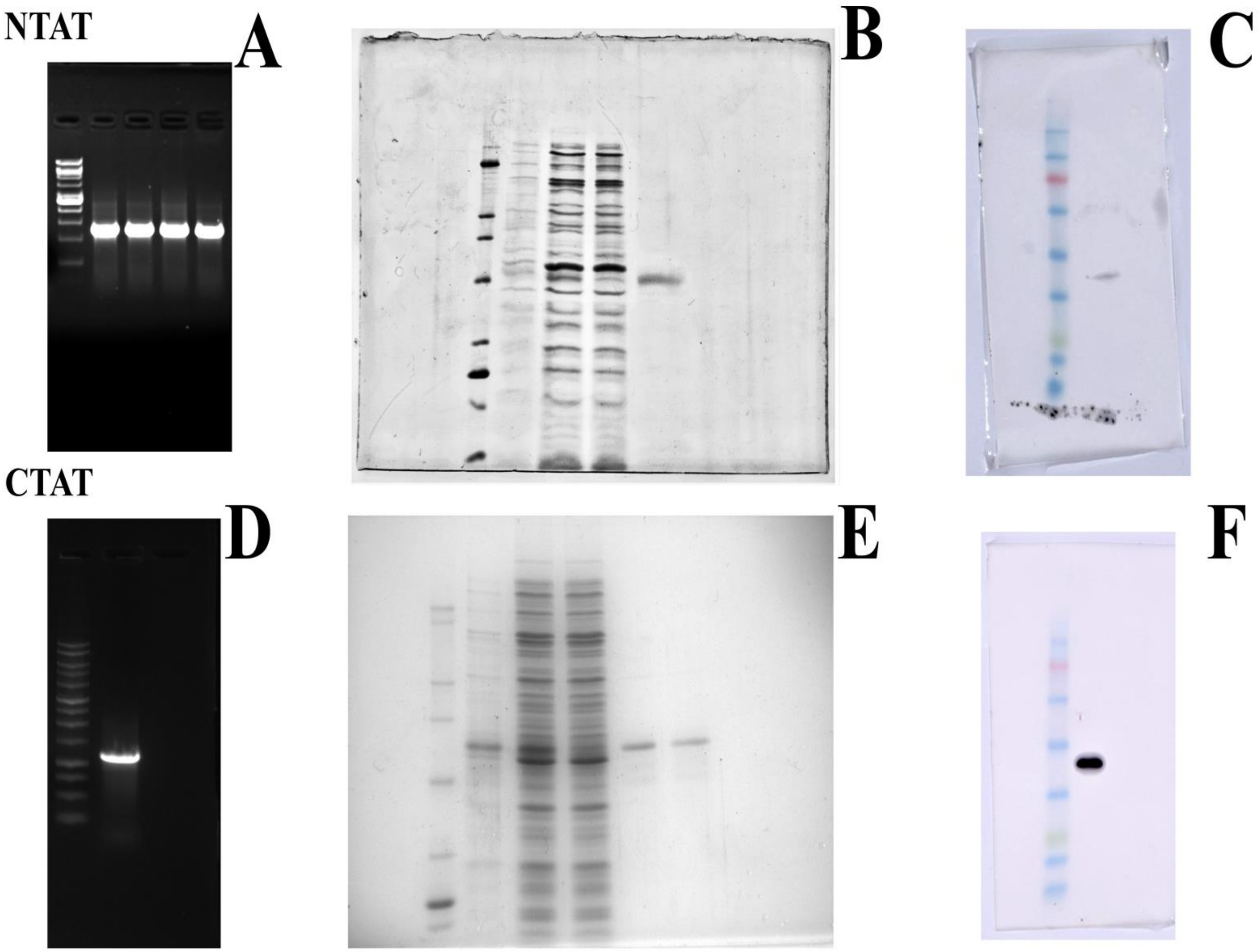

Supplementary Data 5: Uncropped gel images: (A) and (D) Agarose gel electrophoresis images showing NTAT and CTAT respectively **(B) and (E)** SDS-PAGE gel images of NTAT and CTAT respectively **(C) and (F)** Western Blot images of His-tagged NTAT and CTAT respectively.
